# Experiences and Challenges of Implementing Universal Health Coverage With China’s National Basic Public Health Service Program: Literature Review, Regression Analysis, and Insider Interviews

**DOI:** 10.2196/31289

**Published:** 2022-07-22

**Authors:** Guixia Fang, Diling Yang, Li Wang, Zhihao Wang, Yuanyuan Liang, Jinxia Yang

**Affiliations:** 1 School of Health Service Management Anhui Medical University Hefei China; 2 The First Affiliated Hospital University of Science and Technology of China Hefei China; 3 Division of Life Sciences and Medicine University of Science and Technology of China Hefei China; 4 Anhui Provincial Center for Disease Control and Prevention Hefei China

**Keywords:** universal health coverage, basic public health service, China, experience, challenge

## Abstract

**Background:**

Public health service is an important component and pathway to achieve universal health coverage (UHC), a major direction goal of many countries. China’s National Basic Public Health Service Program (the Program) is highly consistent with this direction.

**Objective:**

The aim of this study was to analyze the key experience and challenges of the Program so as to present China’s approach to UHC, help other countries understand and learn from China’s experience, and promote UHC across the world.

**Methods:**

A literature review was performed across five main electronic databases and other sources. Some data were obtained from the Department of Primary Health, National Health Commission, China. Data obtained included the financing share of the national/provincial/prefectural government among the total investment of the program in 32 provinces in 2016, their respective per capita funding levels, and some indicators related to program implementation from 2009 to 2016. The Joinpoint regression model was adopted to test the time trend of changes in program implementation indicators. Face-to-face individual interviews and group discussions were conducted with 48 key insiders.

**Results:**

The program provided full life cycle service to the whole population with an equitable and affordable financing system, enhanced the capability and quality of the health workforce, and facilitated integration of the public health service delivery system. Meanwhile, there were also some shortcomings, including lack of selection and an exit mechanism of service items, inadequate system integration, shortage of qualified professionals, limited role played by actors outside the health sector, and a large gap between the subsidy standard and the actual service cost. The Joinpoint regression analysis demonstrated that 13 indicators related to program implementation showed a significant upward trend (*P*<.05) from 2009 to 2016, with average annual percent change values above 10% for 6 indicators and below 6% for 7 indicators. Three indicators (coverage of health records, electronic health records, and health management among the elderly) rose rapidly with annual percent change values above 30% between 2009 and 2011, but rose slowly or remained stable between 2011 and 2016. In 2016, the subsidy standard per capita in the eastern, central, and western regions was equivalent to US $7.43, $7.15, and $6.57, respectively, of which the national-level subsidy accounted for 25.50%, 60.57%, and 79.52%, respectively.

**Conclusions:**

The Program has made a significant contribution to China’s efforts in achieving UHC. The Program focuses on a key population and provides full life cycle services for the whole population. The financing system completely supported by the government makes the services more equitable and affordable. However, there are a few challenges to implementing the Program in China, especially to increase the public investment, optimize service items, enhance quality of the services, and evaluate the health outcomes.

## Introduction

Countries across the world are making great efforts to achieve universal health coverage (UHC) [1]. In April 2018, at the 70th anniversary of its founding, the World Health Organization (WHO) announced that the tagline for the 69th World Health Day was “Universal health coverage: everyone, everywhere.” To this end, public health is indispensable [2]. The Declaration of Alma-Ata adopted in 1978 defined primary care as essential health care that is universally accessible to all individuals and families in a community [3], highlighting the significance of primary care for UHC [4]. In 2018, the Declaration of Astana reiterated the importance of health promotion and disease prevention, and the role of primary care as a bridge between UHC and public health service in the current context [5]. In China, as a major component of primary care, public health service once made a remarkable contribution to reducing infectious diseases and maternal and pediatric diseases, and promoted the development of primary care globally [6]. China made a commitment to prioritizing UHC in its recent strategic agenda [7], and aligned public health service delivery with efforts toward UHC by the National Basic Public Health Service Program (hereafter referred to as “the Program”) was launched in 2009 [8].

According to the WHO, five key elements are needed to achieve UHC: (1) an efficient, well-run health system that can meet major health needs by providing people-centered health care services to all, particularly pregnant women, children, and people living with HIV/AIDS, tuberculosis, malaria, or noncommunicable diseases (NCDs); (2) a strong health financing system ensuring equitable access to affordable health care services; (3) access to essential medicines, equipment, and technologies for diagnosis and treatment; (4) well-trained and motivated health workers; and (5) recognition that all sectors, inter alia education, transportation, agriculture, and urban construction, have their own roles to play for the health of the population [9]. The Program provides basic public health services to all residents free of charge to address major health challenges among urban and rural citizens, especially children, pregnant women, the elderly, and those with NCDs. The existing service package covers 55 items in 14 categories (see below for details). It is fair to say that the Program has made a significant contribution to China’s efforts toward UHC.

First, the Program targets issues that are highly consistent with the UHC concept. The former covers many priorities of the latter, including vaccination, reporting of and response to communicable diseases and emergency public health events, case management of hypertension and type 2 diabetes, maternal and child health, and health management of the elderly [10]. Second, these services are delivered to all Chinese citizens free of charge, which ensures their affordability and equity [11]. Third, the Program helps to build up the expertise of primary health workers, as they are trained and equipped with adequate technical skills to deliver the services with good quality and high efficiency [12]. The Program also requires the ability to communicate with the population in the catchment, process information, and identify problems. Such nontechnical skills of health workers are also enhanced, as they provide health education, health record management, and follow-up visits. As a result, a more versatile and qualified health workforce is taking shape. Fourth, implementation of the Program contributes to some other building blocks of UHC. For example, health record management requires development of an information system [13]; maternal and child health management and physical checkups require necessary equipment; and health promotion involves collaboration with the sports sector, urban planning institutions, civil affair authorities, and others [14].

Currently, national and international academic communities study UHC from different perspectives. Urquieta-Salomón and Villarreal [15] analyzed the coverage of health insurance and access to preventive medical care, and concluded that interventions on prenatal care and NCDs prevention were greatly inadequate. Atun [16] summarized the experience in achieving UHC by reviewing health insurance reform. Zheng et al [17] elaborated upon China’s health care service delivery system with a focus on the measures for UHC and their outcomes. Shi [18] studied how to achieve UHC in China in terms of concept, policy, and strategy, with little attention paid to prevention and public health. Shan et al [19] and Jiang and Ma [20] identified potential challenges in China’s health insurance on its journey to UHC. Chi et al [21] performed a gray relational analysis to evaluate to what extent UHC has been achieved in China, Germany, Britain, Cuba, and Thailand; the results showed that China was the least close to achieving UHC, and that equity in financing and benefits was a major challenge. Liang and Langenbrunner [22] analyzed the development path of UHC in China from an external perspective, pointing out that there is still a long way to go before China can truly achieve UHC, and put forward suggestions toward achieving this goal, including strengthening disease risk protection, improving health service equity, and improving health system performance. The UHC Monitoring Report released by the WHO in 2019 pointed out that most countries are underinvesting in primary health care and recommended that countries increase spending on primary health care to increase UHC [23].

To date, there have been few studies investigating the relationship between the Program and UHC. However, implementing the Program has become an important pathway and means for China to achieve UHC. Thus, it is necessary and important to analyze its key experience and challenges so as to present China’s approach to UHC, help other countries understand and learn from this experience, and ultimately promote UHC worldwide.

## Methods

### Literature Search

Both the English and Chinese literature was searched on the PubMed, Web of Science, CNKI (China National Knowledge Infrastructure), Wanfang Data, and CQVIP databases with the key words “universal health coverage” and “basic public health service.” Related policy documents and data were also collected from the websites of the National Health Commission (NHC), WHO, and World Bank Database, among others. After eliminating duplicate records, the titles and abstracts of articles with close relevance were thoroughly reviewed by subrelated topics such as “basic public health service projects,” “project financing system,” “health human resource allocation,” and “integration status of public health service system,” and articles with high relevance to these topics were reviewed in full. At the same time, the third edition of the National Basic Public Health Service Specification published on the official website, along with the policy documents, statistical yearbooks, and service guidelines issued by China from 2009 to 2020 to coordinate the implementation of basic public health service projects were collected. The differences in the types, projects, and contents of the services in the three versions of the National Basic Public Health Service Specification were obtained, and the subsidy standards for per capita basic public health services in each year were obtained from the policy documents. These data played a complementary and cross-verification role with the subsequent quantitative data analysis and qualitative interview data, which was conducive to synthesizing all aspects of the information and for performing in-depth exploration and analysis. The specific search strategies in each database are shown in Table 1.

**Table 1:** Literature review search strategies.

Database	Search strings
PubMed	(1) “universal health coverage”[Title/Abstract] AND (“China”[MeSH^a^ Terms] OR “China”[All Fields] OR “China s”[All Fields] OR “Chinas”[All Fields]); (2) “basic public health service”[Title/Abstract]
Web of Science	(1) universal health coverage (subject) and China (subject); (2) basic public health service and China (theme)
CNKI^b^	(1) (Theme: Universal Health Coverage (precise)) AND (Full text: China (precise)); (2) (Theme: “Basic Public Health Services” (Precise)) AND (Theme: Experience + Problem + Challenge (Precise))
CQVIP	(1) Title or keyword=Universal Health Coverage AND any field=China; (2) Title or keyword=Basic public health service AND title or keyword=Experience OR problem OR challenge
Wanfang	(1) Title or keyword: (“Universal Health Coverage”) and All: (China); (2) Title or keyword: (“essential public health services”) and Title or keyword: (Experience or problem or challenge)
Policy document	Basic public health service

^a^MeSH: Medical Subject Heading.

^b^CNKI: China National Knowledge Infrastructure.

### Statistical Analysis

Entrusted by the Department of Primary Health, NHC, we started to evaluate the results of the Program in 2018 and obtained some data, including the financing from the national government as a share of the total investment in the Program in 32 provinces in 2016, their respective per capita funding levels, and some indicators of the Program from 2009 to 2016, such as coverage of health records, coverage of electronic health records (EHRs), utilization rate of health records, amount of printed health education materials distributed per 10,000 people, number of participants of health education activities per 10,000 people, share of children with a vaccination record book, share of children receiving standard Expanded Program on Immunization (EPI) vaccination, rate of newborn home visits, coverage of health management among children aged 0-6 years, rate of registration in early pregnancy, coverage of postnatal visits, coverage of health management among the elderly, percentage of patients with hypertension under standardized management, percentage of patients with type 2 diabetes under standardized management, and coverage of health management by traditional Chinese medicine (TCM). The data were analyzed in Microsoft Office Excel 2010 for Windows (Microsoft Corp). The data were initially collected by the provincial health commission and then reported to the NHC (as China’s top-level basic health management authority) before finally reaching us. With an official check by the above-mentioned authorities, the accuracy and reliability of all the data are highly guaranteed.

To test the changes of time trend in the basic public health service project, the Joinpoint regression program version 4.9.0.0 (National Cancer Institute) was adopted to analyze and determine the year of significant changes. The Joinpoint regression model was adopted to evaluate the average annual percentage change (AAPC) and annual percentage change (APC) with the corresponding 95% CIs in each time trend segment.

### Interviews With Key Insiders

The interviewees included policymakers, frontline workers, and other relevant personnel who have been engaged in China’s public health work for many years. Face-to-face individual interviews and group discussions were conducted. The 48 interviewees included one representative from each of the three national-level institutions, namely the Department of Primary Health of the NHC, National Center for Disease Control (CDC), and the National Disease Prevention and Control Bureau of the NHC; three leaders and three staff from the Primary Health Division of the provincial-level health commission who were responsible for the Program; three from the provincial-level CDC; three county hospital presidents and three public health workers in county hospitals; three township health center (THC) directors and three public health workers in THCs; nine village doctors; three directors of community health care centers (CHCs), which are in urban areas, and three public health workers in CHCs; and nine doctors from CHC stations. Provincial-level personnel and grassroots personnel came from Liaoning, Anhui, and Chongqing, representing China’s eastern, central, and western regions, respectively. All of the interviewees have good knowledge about the practices related to UHC and basic public health service in China.

The full process was recorded, and transcripts were noted for the classification and analysis by topics.

The main interview questions were as follows:

1. Is the local basic public health service information platform connected with other systems?

2. What agencies/departments have you cooperated with or contacted while carrying out basic health service projects locally?

3. How well do you think residents are accepting basic public health services?

4. What role do you consider the implementing basic public services play to advance universal health coverage?

5. What problems do you think exist in the implementation of basic public health service projects?

### Ethics Considerations

Verbal informed consent was received in all interviews. This study was approved by the Biomedical Ethics Committee of Anhui Medical University (code 20150082).

## Results

### Achievements of the Program

#### The Program Provides a Full Life Cycle Service to the Whole Population

After the Program was launched in 2009, the National Health and Family Planning Commission issued three editions of the National Basic Public Service Specifications in 2009, 2011, and 2017, respectively. The service package expanded from 41 items in 9 categories in 2009 to 55 items in 14 categories in 2017. Regardless of the specific changes to the service items, the Program has always covered the entire life cycle and the whole population, especially pregnant women, children, the elderly, and those with NCDs or tuberculosis (Table 2).

**Table 2:** Changes in the service categories among the three editions of the National Basic Public Service Specifications.

Edition (year)	Categories of the service	Changes
First edition (2009)	10 categories: (1) health record management for urban and rural residents; (2) health education; (3) health management of children aged 0-6 years; (4) maternal health management; (5) elderly health management; (6) vaccination; (7) reporting of and response to communicable diseases; (8) management of patients with hypertension; (9) management of patients with type 2 diabetes; and (10) management of patients with severe mental illness	Not applicable
Second edition (2011)	11 categories: (1) health record management for urban and rural residents; (2) health education; (3) vaccination; (4) health management of children aged 0-6 years; (5) maternal health management; (6) elderly health management; (7) management of patients with hypertension; (8) management of patients with type 2 diabetes; (9) management of patients with severe mental illness; (10) reporting of and response to communicable diseases and public health emergencies; and (11) health inspection and coordination	“Reporting of and response to communicable diseases” was changed to “reporting of and response to communicable diseases and public health emergencies,” and “health inspection and coordination” was added
Third edition (2017)	14 categories: (1) health record management for urban and rural residents; (2) health education; (3) vaccination; (4) health management of children aged 0-6 years; (5) maternal health management; (6) elderly health management; (7) management of people with NCDs^a^ (including patients with hypertension and patients with type 2 diabetes); (8) management of patients with severe mental illness; (9) management of patients with TB^b^; (10) health management by TCM^c^; (11) reporting of and response to communicable diseases and public health emergencies; (12) health inspection and coordination; (13) free contraceptives; and (14) health promotion	“Management of patients with hypertension” and “management of patients with type 2 diabetes” were merged into “management of people with NCDs”; new categories include “management of patients with TB,” “free contraceptives,” “health management by TCM,” and “health promotion”

^a^NCD: noncommunicable disease.

^b^TB: tuberculosis.

^c^TCM: traditional Chinese medicine.

Some indicators showed that the coverage of the Program had gradually expanded to the whole population. In addition to the rising share of citizens with health records and higher utilization rate of those records, the share of children with a vaccination record book, share of children receiving standard EPI vaccination, and coverage of maternal and child health management were all above 90%. Coverage of elderly health management was approximately 70%. The share of patients with NCDs under management, along with the control rate of blood pressure and glucose, was on the rise. Since 2012, health management by TCM has developed rapidly. All of these facts indicated that the Program provided UHC services to hundreds of millions of people (Table 3).

The results of Joinpoint regression analysis are presented in Table 4. Thirteen indicators relating to the implementation of the program showed a significant upward trend (*P*<.05) from 2009 to 2016, including coverage of EHRs, coverage of health management among the elderly, and glucose control rate among the managed patients with type 2 diabetes, whereas there was no significant change in the share of children with a vaccination record book, share of children receiving standard EPI vaccination, and percentage of patients with hypertension under standardized management (all *P*>.05). The AAPC values of six indicators, including coverage of health records, coverage of EHRs, utilization rate of health records, coverage of health management among the elderly, coverage of health management by TCM among the elderly, and coverage of health management by TCM among children aged 0-36 months, were all above 10%, and those of the other seven indicators, including the rate of newborn home visits, coverage of health management among children aged 0-6 years, and rate of registration in early pregnancy, were all below 6%. The coverage of health records, EHRs, and health management among children aged 0-6 years rose rapidly between 2009 and 2011, but rose slowly between 2011 and 2016. The coverage of health management among the elderly increased significantly between 2009 and 2011, but remained stable between 2011 and 2016, and the glucose control rate among the managed patients with type 2 diabetes increased significantly between 2009 and 2013, but remained stable between 2013 and 2016.

**Table 3:** Representative indicators on implementation of the Program 2009-2016.^a^

Indicators	2009	2010	2011	2012	2013	2014	2015	2016
Coverage of health records, %	33.38	49.12	64.31	73.33	79.81	82.35	84.97	88.56
Coverage of electronic health records, %	17.64	28.40	58.99	69.62	76.36	78.01	81.65	85.46
Utilization rate of health records, %	14.22	14.80	17.66	22.34	29.95	46.98	51.4	55.11
Hard copies of print health education materials distributed per 10,000 people, n	1402.04	1613.33	5404.75	4501.36	4205.84	4845.88	4819.35	4408.12
Participants of health education activities per 10,000 people, n	387.20	570.87	1030.72	1487.17	1473.60	1001.92	1044.09	938.24
Share of children with a vaccination record book, %	99.73	98.91	99.46	99.71	99.63	99.71	99.85	99.97
Share of children receiving standard EPI^b^ vaccination, %	98.19	98.93	99.32	99.01	99.29	99.33	99.44	99.14
Rate of newborn home visits, %	81.80	81.55	87.32	89.66	90.39	92.44	93.83	94.77
Coverage of health management among children aged 0-6 years, %	73.41	80.47	85.33	88.64	89.87	90.85	91.6	91.82
Rate of registration in early pregnancy, %	78.62	80.62	83.01	86.76	87.53	89.20	91.57	91.42
Coverage of postnatal visits, %	87.33	85.27	89.11	90.83	91.83	92.95	94.30	94.41
Coverage of health management among the elderly, %	35.17	50.59	65.21	71.80	70.03	72.47	73.29	70.74
Share of patients with hypertension under standardized management, %	68.96	70.77	68.29	70.56	69.28	74.67	70.03	71.64
Blood pressure control rate among the managed patients with hypertension, %	40.68	50.52	48.92	49.96	56.51	59.23	60.96	62.40
Share of patients with type 2 diabetes under standardized management, %	69.58	71.19	68.88	69.54	71.48	75.25	73.01	74.58
Glucose control rate among the managed patients with type 2 diabetes, %	40.03	40.18	45.88	48.54	56.16	55.63	54.25	57.93
Coverage of health management by TCM^c^ among the elderly, %	13.85	16.42	18.05	16.86	26.13	37.87	46.98	53.23
Coverage of health management by TCM among children aged 0-36 months, %	13.79	15.54	17.16	18.14	27.15	41.87	48.27	55.69

^a^Source: National Survey on Primary Health Facilities 2016 by the Department of Primary Health, National Health Commission, China.

^b^EPI: Expanded Program on Immunization.

^c^TCM: traditional Chinese medicine.

**Table 4:** Joinpoint regression analysis of trends for representative indicators on the implementation of the Program, 2009-2016.

Indicators	AAPC^a^, % (95% CI)	*P* value	Trend^b^ 1				Trend 2		
			Period	APC^c^, % (95% CI)	*P* value	Period		APC, % (95% CI)	*P* value
Coverage of health records, (%)	15.0 (11.7 to 18.4)	<.001	2009-2011	42.1 (21.0 to 66.8)	.006	2011-2016		5.7 (3.8 to 7.5)	.002
Coverage of electronic health records (%)	28.1 (21.5 to 35.0)	<.001	2009-2011	102.9 (51.1 to 172.4)	.005	2011-2016		6.5 (4.6 to 8.5)	.002
Utilization rate of health records (%)	23.7 (15.6 to 32.3)	<.001	2009-2016	23.7 (15.6 to 32.3)	<.001	N/A^d^		N/A	N/A
Share of children with a vaccination record book (%)	0.1 (–0.0 to 0.2)	.09	2009-2016	0.1 (–0.0 to 0.2)	.09	N/A		N/A	N/A
Share of children receiving standard EPI^e^ vaccination (%)	0.1 (–0.0 to 0.3)	.09	2009-2011	0.5 (–0.3 to 1.3)	.15	2011-2016		0.0 (–0.2 to 0.2)	.99
Rate of newborn home visits (%)	2.3 (1.6 to 2.9)	<.001	2009-2016	2.3 (1.6 to 2.9)	<.001	N/A		N/A	N/A
Coverage of health management among children aged 0-6 years (%)	3.3 (2.9 to 3.8)	<.001	2009-2011	8.6 (6.3 to 10.9)	.001	2011-2016		1.3 (0.9 to 1.7)	.002
Rate of registration in early pregnancy (%)	2.3 (1.8 to 2.8)	<.001	2009-2016	2.3 (1.8 to 2.8)	<.001	N/A		N/A	N/A
Coverage of postnatal visit (%)	1.4 (0.9 to 1.9)	<.001	2009-2016	1.4 (0.9 to 1.9)	<.001	N/A		N/A	N/A
Coverage of health management among the elderly (%)	10.7 (7.4 to 14.1)	<.001	2009-2011	38.2 (17.2 to 62.9)	.008	2011-2016		1.3 (–0.8 to 3.4)	.15
Share of patients with hypertension under standardized management (%)	0.6 (–0.5 to 1.6)	.22	2009-2016	0.6 (–0.5 to 1.6)	.22	N/A^d^		N/A	N/A
Blood pressure control rate among the managed patients with hypertension (%)	5.3 (3.4 to 7.3)	<.001	2009-2016	5.3 (3.4 to 7.3)	<.001	N/A		N/A	N/A
Share of patients with type 2 diabetes under standardized management (%)	1.1 (0.3 to 1.9)	.02	2009-2016	1.1 (0.3 to 1.9)	.02	N/A		N/A	N/A
Glucose control rate among the managed patients with type 2 diabetes (%)	5.8 (3.0 to 8.6)	<.001	2009-2013	9.2 (3.3 to 15.4)	.02	2013-2016		1.4 (–5.3 to 8.5)	.57
Coverage of health management by TCM^f^ among the elderly (%)	24.1 (18.5 to 30.1)	<.001	2009-2016	24.1 (18.5 to 30.1)	<.001	N/A		N/A	N/A
Coverage of health management by TCM among children aged 0-36 months (%)	25.1 (18.8 to 31.8)	<.001	2009-2016	25.1 (18.8 to 31.8)	<.001	N/A		N/A	N/A

^a^Trend: two temporal trend segments (Trend 1 and Trend 2) that join up at one join point recognized by the Joinpoint regression model.

^b^AAPC: average annual percent change.

^c^APC: annual percent change.

^d^N/A: not applicable.

^e^EPI: Expanded Program on Immunization.

^f^TCM: traditional Chinese medicine.

#### The Financing Mechanism of the Program Ensures Equity and Affordability

Since 2009, China has strived for the gradual equalization of basic public health services [24]. To this end, the Program is completely financed by governments at all levels, including the national, provincial, and prefecture levels and below. Due to regional economic disparity, government fiscal strength among provinces varies greatly. To ensure the equitable access to basic public health services, the national government requires that actual per capita financing for the Program should not be lower than the national standard. For example, in 2016, the state required that the per capita subsidy standard for basic public health service projects should not be less than the equivalent of US $6.51 (1 Yuan Renminbi=US $0.15 in 2016) and the result was that the per capita subsidy standard in the eastern, central, and western regions was US $7.43, $7.15, and $6.57, respectively, of which the national-level subsidy accounted for 25.50%, 60.57%, and 79.52%, respectively (see Table 5 for details). This means that according to the actual situation of economic development, the central government will give more financial support to the underdeveloped provinces in the west through transfer payments. The total public investment in the Program increased from US $2.4 billion in 2009 to US $9.6 billion in 2016 [25]. The per capita standard rose from US $2.20 in 2009 [26] to US $11.16 in 2020 [27], and will definitely continue to grow. Such a financing mechanism recognizes regional gaps and guarantees equity and affordability, therefore helping to provide better access to health services [28].

**Table 5:** Fiscal investment in the Program by regions for 2016.

Financing standard	Eastern region (Yuan^a^), average (% share)	Central region (Yuan), average (% share)	Western region (Yuan), average (% share)
Per capita standard	51.32 (100.00)	49.38 (100.00)	45.42 (100.00)
By national budget	13.09 (25.50)	29.91 (60.57)	36.12 (79.52)
By provincial budget	8.98 (17.50)	8.51 (17.23)	5.36 (11.80)
By prefectural budget and below	29.25 (57.00)	10.96 (22.20)	3.94 (8.68)

^a^1 Chinese Yuan Renminbi=US $0.1447 in 2016.

#### Implementation of the Program Enhances Capability and Quality of the Health Workforce

Primary health workers are the mainstay to deliver basic public health services and a major contributor to UHC. To achieve the goals, competent departments provide them with targeted training to improve their capability and quality. For instance, health authorities introduced a package of policies such as the Program of Strengthening Rural Healthcare Service Delivery Network to enhance their management and technical skills through training [29]. In addition, since the new round of health care reform was launched in 2009, the central government has asked THCs to design well-structured and targeted training programs to update village doctors’ knowledge and skills in public health services [30].

Local governments organize various training on the policies, technical protocols, knowledge, and skills related to the Program for those who manage or implement it, including public health managers, professionals, and village doctors. In some places, they also implement contests on the Programs and exams on the technical protocols of the services. In other provinces, ear-marked transfer from the national level is used for capacity building of CHC health workers. Other activities include a contracted-based education program of public health doctors or village doctors, which requires trainees to work at a primary level for a certain period after graduation, further education for the village doctors who had only received secondary vocational training, and training to upgrade village doctors into licensed assistant doctors [31].

In general, the Program improves the health workers’ capability and quality in three areas. First, they are more capable of providing integrated care. The Program is established to deliver integrated care, including both preventive and curative interventions. This requires the services of primary health workers to shift from a disease-centered to a health-centered model [32]. Second, as the services covered by the Program are all delivered at the primary level, primary health workers have direct contact with local residents and need to maintain a good relationship with them [33]. More attention is given to communication skills, patience, and empathy. Third, they are better at identifying problems during service delivery; managing, analyzing, and utilizing information; and diagnosing and addressing regional health issues [34]. Some studies found that the theoretical knowledge and practical skills of the trained primary health workers had improved greatly [35-38].

#### The Program Facilitates the Integration of a Public Health Service Delivery System

Previously, China’s public health service delivery system was fragmented, composed of many specialist public health institutions responsible for health education, disease prevention and control, endemic and occupational disease prevention, and maternal and child health care, respectively. Primary health care institutions were not included in the system. However, the Program was mainly implemented by primary institutions, along with participation by these specialist institutions through technical assistance, technical training, and development of protocols. Various players in the system work together. They complement, collaborate with, and support each other, which gradually integrate the public health service delivery system [39]. This is a significant step forward to achieve integrated continuous care as well as UHC.

### Shortcomings of the Program

#### No Well-Established Selection and Exit Mechanism for Service Items, and the Number of Items Keeps Rising

First, the service package of the Program cannot meet the actual needs of the general public. This is largely associated with the fact that service items are set based on how much public financing is available [40]. UHC aims to provide people-centered, quality services to satisfy the needs of the population. However, with higher health literacy, the needs are growing and becoming more diversified. Moreover, public health priorities are continuously changing over time, which requires timely updates of the service package [41].

Since the Program covers a large number of services and a huge target population, primary health workers can only manage to hit the set target in service volume but must compromise on quality. Indeed, the services sometimes fail to live up to the standards set in the Basic Public Health Service Specification (3rd edition) [42]. For example, some follow-ups fall short of requirement of the Specification. In some places, providers may only adopt the service as a matter of formality. While service volume and items increase continuously, the conflict between the actual needs of citizens and the service capacity of the primary level is not taken into consideration adequately. For instance, two new items, health management by TCM and management of patients with severe mental illness, were included in the package recently; however, primary institutions do not have the capability of delivering these services. Without sufficient knowledge and skills in TCM, village doctors have difficulties in carrying out TCM interventions. As a result, the pressure of health workers mounts on the one hand and citizens are not satisfied with the services on the other hand, which negatively affects the result of the Program.

#### Poor Integration of the Service Delivery System Lowers the Efficiency

Second, poor integration among the service items lowers the efficiency of the system. There are many service items in the package but little integration among them. For example, health records for the whole population are isolated from maternal, child, and elderly health management, and NCD cases are managed separately according to conditions [43]. All of these aspects result in unnecessary duplication in terms of effort and systems. Moreover, basic public health services and essential medical care are not well connected [44]. Taking NCD management as an example, services by general practitioners (GPs), specialists, and public health workers are so poorly integrated that a closed-end service network is absent, which has a negative influence on the quality of NCD management.

Furthermore, the information technology (IT) system is fragmented and poorly integrated [45]. Without a single top-level framework, IT systems are not compatible and connected with each other, which cannot meet the needs of the Program. There are nationwide systems for vaccination, management of patients with severe mental illness, and communicable disease reporting, as well as province-wide systems for maternal and child care. For the remaining items, there are few province-wide or prefecture-level information systems. In most cases, it is the counties/districts that authorize IT companies to develop the systems needed, which wastes substantial human and financial resources. Li et al [6] showed that the IT systems of primary health institutions with in-house EHRs were developed by over 80 IT providers independently with little connectivity and interoperability. Poor integration among these systems hinders information sharing across institutions and regions, which impacts the result of the Program [46]. For instance, although health records cover the majority of the population, many of them are not sufficiently active [47]. Moreover, the isolation between health record systems and the systems of health institutions hampers the integration of preventive and medical care.

#### Shortage of Well-Trained Health Professionals

The third challenge is insufficient well-trained primary health workers, who are the main implementers of the Program. According to the Statistic Bulletin on China’s Health Sector 2018, health workers in THCs and CHCs only account for 32.2% of the national total [48]. GPs, representing 8.49% of all doctors, represent the profession that faces the greatest shortage. At the end of 2018, the number of GPs per 10,000 people was only 2.22, which is still lower than the upper-end of target for 2020 of 2–3 GPs per 10,000 citizens [49].

The increasing workload on health workers also highlights the understaffed issue in health institutions. As service items and the population served are both on the rise, it is common that one primary health worker takes on the responsibilities of several. A survey among primary health workers in three provinces showed that 27.1% considered that after implementation of the Program, the workload increased to an unbearable level [50]. Shi et al [51] indicated that 4307 out of 10,626 (40.53%) primary care physicians were extremely tired. Li et al [52] found that 627 of 1221 (51.35%) village doctors in three provinces were not satisfied with the situation. Sun et al [53] found that 1244 of 3212 (38.73%) CHC health workers in five provinces wanted to quit the job. Meanwhile, given the restriction in the current recruitment system and lack of supportive measures, it is very hard to effectively replenish the primary health workforce. Moreover, primary health workers are often less knowledgeable with a lower education level and professional title [54]. Among staff of CHCs and THCs, those with an associate degree and below account for 71.9% and 91.3%, respectively. Village doctors are even more poorly educated [55]; 21% of primary health professionals are not licensed physicians or licensed assistant physicians [6]. Lack of human resources and inadequate service capability have greatly impacted implementation of the Program.

#### Government Agencies Outside the Health Sector Are Rarely Engaged and Interdepartment Collaboration is Weak

Fourth, China’s basic public health service delivery system remains an isolated island. The services are mainly delivered by health institutions. Local governments usually do not take the lead and deliver due responsibility in the Program implementation, because the basic public health service is often considered to be the mandate of the health sector only, and thus has little to do with the government as a whole [56]. Therefore, other government agencies are poorly motivated to engage in this area. However, many of them, such as public security, civil affairs, neighborhood committees, and health insurers, should play important roles in information sharing and promotion campaigns, while primary health institutions function as the service providers. In practice, it is always the latter that propose collaboration with other agencies. In some cases, the public security department might even refuse to share information of the population in the catchment under the excuse of confidentiality. This is, to a large extent, because departments cannot reach a consensus on their cooperation in the absence of incentives or institutionalized operable binding mechanisms for coordination [57].

#### Per Capita Public Financing Varies Among Regions and is Lower Than the Cost

Fifth, public financing varies among regions, which may lead to inequity in health outcomes. In most provinces, underdeveloped provinces in particular, public financing for the Program follows the national standard and 80% of the financing is derived from transfer payments by the central government. However, in developed provinces, especially in the eastern region, the per capita financing is more than the national standard and service packages are more generous than the national package, because provincial and municipal governments allocate more investment to the Program. In 2016, 24 out of the 32 provinces (autonomous regions and municipalities directly under the central government) followed the national financing standard (US $6.51 per capita), and in the other 8 provinces, the financing was higher than the national standard (eg, US $19.68 per capita in Beijing and US $11.14 per capita in Shanghai) [56]. Unsurprisingly, in the areas where the per capita standard is much higher than the national standard, local residents enjoy better services. Health inequity derived from the regional economic gap is a stark fact, which is inconsistent with UHC [58].

In the exiting financing mechanism, a national standard guarantees basic equity among regions. However, in practice, provinces and regions with a strong economy have more financial and material resources available to finance the Program, while underdeveloped regions cannot afford the Program, even though their needs in disease prevention and control are more urgent. This results in a regressive effect, and widens the gap in public health service between the rich and poor regions [59].

In addition, there is an increasingly large gap between the financing standard and the actual service cost, which threatens the sustainability of the Program. With the growing demand for health, Chinese people, especially those living in cities and economically developed areas, want not only a longer life but also a healthier one, and their demand for basic public health service is increasing. For example, the elderly want their physical checkups to be as comprehensive as those performed in large hospitals, covering more complicated items such as computed tomography scans. Patients with hypertension or diabetes would like to receive imported drugs, which also comes with a higher cost. For instance, a complex package of lab tests will cost health institutions US $15. In practice, the Program was designed to provide universally accessible basic public health services. With a high-cost package in place, local financing is stressful, and primary health institutions have to finance public health services by the income from the medical service to reach the set target. Moreover, due to the geographical environment, customs, and other factors in different regions, the service delivery cost varies greatly.

## Discussion

### Achievements and Shortcomings of Program Implementation

The comprehensive promotion of the Program and the rapid development of the universal medical insurance system are two important measures that have jointly affected UHC in the New Medical Reform, which has attracted worldwide attention since 2009. These measures serve to build up a UHC system integrating preventive and curative care. Since its implementation, the Program has played a positive role in providing life cycle public health services for the whole population; enhancing equity, accessibility, and affordability of the public health service; improving the quality of the primary health workforce; and integrating the public health service delivery system. However, at the same time, there are still some challenges to overcome, such as a rigid item inclusion and exclusion mechanism that is irresponsive to new developments, overemphasis on prevention but neglect of medical care, backward and fragmented information systems, insufficient human resources, and inadequate government functioning.

In the Program, five categories of services, including health record management for urban and rural residents, elderly health management, management of patients with hypertension, management of patients with type 2 diabetes, and health management by TCM, are considered to be developing from scratch in China. Among the 10 indicators relating to these services, except for the percentage of patients with hypertension under standardized management, the remaining 9 indicators have increased significantly, especially the 6 indicators relating to health record management for urban and rural residents, health management by TCM, and elderly health management, which increased at a relatively higher rate. Two categories of services (health management of children aged 0-6 years and maternal health management) had been implemented for a longer period of time before the Program was established and were thus relatively mature; consequently, all 4 indicators relating to these services rose relatively slowly. The vaccination service has been carried out in China for decades, and it is so widely accepted that the two indicators relating to this type of service have remained at about 99% after the implementation of the Program, with no room for growth.

Five indicators, including coverage of health records, coverage of EHRs, health management among children aged 0-6 years, health management among the elderly, and glucose control rate among the managed patients with type 2 diabetes, appeared in 2011 or 2013, rose rapidly before the connection point, and then the increase slowed down or tended to be stable after the connection point, which may be related to the increase both in the number of service items and in the workload of grassroots personnel after 2011 [60]. According to the requirements of the National Basic Public Health Service Specification, the standardized management of patients with hypertension and type 2 diabetes should be a follow-up 4 times a year. Between 2009 and 2016, the standardized management rate of these two types of patients was between 68% and 76%; the percentage of patients with hypertension under standardized management did not increase significantly and the percentage of patients with type 2 diabetes under standardized management rose slowly, which may be related to the low compliance of the two types of patients with follow-up services and population flow [61]. In addition, with the popularity of sphygmomanometers and blood glucose meters, coupled with the limited service capacity of grassroots medical personnel, simple follow-up is no longer attractive to patients with type 2 diabetes or hypertension [62].

### International Experience With UHC

Experience from other countries demonstrates that a health system with universal coverage is the foundation of the health sector. Since 2015, advocated by the WHO and United Nations (UN) Assembly, UHC has become a global goal. To this end, countries adopt different measures based on their own context and development stage but also face some common challenges.

The United Kingdom is a frontrunner in UHC; its National Health Service (NHS) provides high-quality health care services to British citizens under the principle of universal access, free care, and needs-based service. This is largely achieved owing to its strong funding system. The NHS budget accounts for 9.6% of the gross domestic product (GDP), whereas China’s health expenditure only accounts for up to 5.6% of its GDP. In addition, 82% of NHS expenditure is funded by the general taxation revenue, only 2% is funded by individual copayment, and the rest is funded by national insurance and donations. This system maximizes the access to health services. However, it shares the same challenges as faced by the Program. For example, each GP in the NHS is responsible for 1500 patients, and the income of physicians has nothing to do with their workload, which leads to low efficiency of the health service delivery system and very long waiting lists [63]. In China, the implementation of the Program also brings about an extremely heavy workload to primary health workers but lacks effective incentives. Germany has also achieved a high level of UHC, where legislation of the health insurance system plays a critical role. The Statutory Health Insurance is the mainstay of Germany’s health insurance system, covering 90% of the population. Such high coverage is partly attributed to the arrangement that the premium covers spouses and children who have no income [64]. The system is operated by a third party selected through a competitive process, which contributes to gains in service quality and efficiency. As a low- and middle-income country, Cubans have good health status, which may be greatly associated with the country’s system of free health care for all. The system guarantees the equity of health insurance and benefits. Thailand’s “30 Baht Plan” expands the health insurance system to the uncovered, which accounts for approximately 30% of the population, so that they have access to health care. As a result, Thailand has officially achieved UHC [65]. The Thai health insurance system is pooling at a national level with better capacity of risk resistance. In contrast, the three major insurance schemes (which are integrated with each other) in China are mostly pooling at the prefectural levels, and even at the county level in some cases. Although the above institutional arrangements in these countries are not specific to the public health service, service items related to prevention, health management, and rehabilitation are all covered by the free service package or the benefit package of the health insurance system.

### China’s Experience With UHC

Countries such as the United Kingdom, Germany, Cuba, Thailand, and others achieve UHC by following a slightly different approach from that adopted by China. Most of these countries do not consider a public health review service as an independent system, but rather cover this service in the health insurance system. By contrast, China defines a basic public health service package with universal coverage, which only includes preventive care and health management [66]. This approach draws attention to prevention and public health, which had been neglected for a long time; facilitates a strong organizational system, logistic safeguard, and political will for its financing and service delivery; and covers the whole population within a short time frame. However, this system also introduces new fragmentation between medical and preventive care. In some cases, the original treatment-centered model shifted to the other extreme: prevention is overemphasized and treatment is neglected. In terms of financing, the public health system is completely independent from the health insurance system; the latter only covers treatment, while the former only focuses on prevention. Such fragmentation is not conducive to the integration of the two and can delay progress in achieving UHC [67].

### Suggestions to Promote UHC in China

Besides financing, the service delivery system for prevention is also relatively independent from that used for curative care, which has become the bottleneck for quality gains at the primary level and the health status of the population [66]. The health care under UHC requires a shift to an integrated service delivery system featured by primary care as its core, better coordination and cooperation among service providers, as well as continuity and integration of services [67]. Better integration can boost the efficiency of the system. South Africa has made impressive progress in primary care largely owing to its focus on integrated care [68]. Erondu et al [2] also appealed that UHC requires a more integrated health system, including public health.

Fang et al [69] argued that without efficiency gains in the health care system, some measures such as strengthening primary care and increasing investment can only bring moderate improvements in financial protection, one of the key objectives of UHC [70].

From a broader perspective, the basic public health service needs to connect with other health-related factors. “Health in All Policies” (HiAP) has become an important guideline for China’s efforts in the health sector in recent years [71]. HiAP is also described as a necessary component of primary care [72]. The Program has started to recognize the negative impact of unhealthy lifestyles, but it is not sufficient to only change individuals’ lifestyle. Social and environmental factors bearing on health should receive more attention. For example, people can have a healthy lifestyle and dietary structure, but cannot avoid the negative impacts due to inhaling polluted air during outdoor exercises or the intake of antibiotics, steroids, and pesticide residues in the diet [71]. The Program should seize the opportunity to work with more partners, especially players in nonhealth sectors, to address policies and structural factors rather than only focusing on individual factors [73].

The Program, with ear-marked funding and specific governance arrangement, has been implemented in full swing very quickly, with some compromise on service quality to a certain extent [66]. At the initial stage, the performance indicators were mainly related to volume, such as the number of records on follow-up visits and coverage of health records, rather than incentives for better quality. The latest study indicates that quality of care, and not only accessibility, is the key determinant to improve population health and reduce the disease burden [74]. For instance, South Africa, where remarkable progress has been made in UHC, takes quality as the core of its efforts [71].

The quality of the basic public health service is also closely related to the existing financing methods and overall health system in China. On the one hand, despite continuous growth of public investment, the financing standard is still too low to satisfy the public expectation of the public health service. Due to regional economic disparity and a decentralized fiscal system, local governments’ expenditure on public health varies greatly, leading to a quality gap among regions. The Outline of the Planning for National Healthcare Service Delivery System (2015-2020) clearly points out that in western China, low-quality health resources and insufficient service capacity at the primary level affect the equity and efficiency of health service [75]. On the other hand, China’s health system remains hospital-centered, and patients tend to go to the higher-level providers for health services. From 2005 to 2015, the proportion of outpatient care provided at the primary level declined by 7% [76], although “strong primary providers” had been continuously emphasized since the launch of the reform in 2009. This is a problem worth pondering. When patients are “voting with their feet,” high-quality human resources are siphoned from the primary level, which aggravates the shortage of primary health workers, especially those of high quality. In 2010, only 5.6% of THC doctors had a 5-year or above medical education. This proportion increased very slowly in the following years, reaching 10% in 2017 [77]. As the capacity of health professionals undoubtedly influences service quality, it is hard to assure the quality of basic public health services delivered at the primary level [78]. At present, China is trying to change the medical education system and education model. The health workforce is expected to improve in the future. However, it remains a significant challenge to ensure the availability of adequate, high-quality health professionals at the primary level [36].

The UN Sustainable Development Goals require all countries to achieve UHC by 2030, focusing on two priorities in particular: universal coverage of essential health services and financial protection [70]. China has made substantial progress toward the first priority, largely due to implementation of the Program and the rapid expansion of health insurance system. However, it still has a long way to go in the second area because of gaps in health financing, service quality, and coordination within the system.

### Conclusion

Based on a review of the implementation of basic public health service projects in China, this study systematically analyzed the role and challenges of the implementation of basic public health services in China, a country with a population of 1.4 billion, to achieve UHC. This experience can also serve as a reference for other countries. The services in the package focus on key populations and provide full life cycle services for the whole population. The financing system completely supported by the government makes the services more equitable and affordable. However, health system reform is complicated and full of daunting challenges, especially for a populous, developing country such as China. In the future, the Program should aim to first balance the relationship between curative and preventive care, and adjust its incentive mechanism. Meanwhile, a continuous increase in public investment is the key for both the health insurance system and to achieve equal access to basic public health services. A policy opportunity is to facilitate the integration of the Program and clinical care, which means a “family doctor team” made up of clinical physicians and public health workers that manages the health of the population in the catchment. Under this model, the continuity and quality of NCD management can be improved and the Program can directly contribute to better health outcomes. At present, many far-sighted experts are working on restructuring China’s service delivery system, and public health should be an important component. The restructuring should follow the principle of HiAP, and aim for a people-centered, community-based, and coordinated service delivery system, which can provide continuous, accountable, and integrated care to satisfy the needs of the population.

## Abbreviations

AAPC: average annual percent change
APC: annual percent change
CDC: Center for Disease Prevention and Control
CHC: community health center
CNKI: China National Knowledge Infrastructure
EHR: electronic health recorded
EPI: Expanded Program on Immunization
GDP: gross domestic product
GP: general practitioner
HiAP: Health in All Policies
IT: information technology
NCD: noncommunicable disease
NHC: National Health Commission
NHS: National Health Service
TCM: traditional Chinese medicine
THC: township health center
UHC: universal health coverage
UN: United Nations
WHO: World Health Organization
